# Improving the Rural-Urban Balance in Cambodia’s Health Services

**DOI:** 10.34172/ijhpm.2020.136

**Published:** 2020-07-26

**Authors:** Yurie Kobashi, Kimhab Chou, Novy Slaiman, Phannoch Neun, Yoshifumi Hayashi, Masaharu Tsubokura, Manabu Okawada

**Affiliations:** ^1^Department of General Internal Medicine, Sunrise Japan Hospital Phnom Penh, Phnom Penh, Cambodia.; ^2^Department of Public Health, Fukushima Medical University School of Medicine, Fukushima, Japan.; ^3^Department of Medicine, University of Puthisastra, Phnom Penh, Cambodia.; ^4^Department of Neurosurgery, Sunrise Japan Hospital Phnom Penh, Phnom Penh, Cambodia.; ^5^Department of Pediatrics, Sunrise Japan Hospital Phnom Penh, Phnom Penh, Cambodia.

## Dear Editor,

A qualified health workforce that is available, equitably distributed and accessible to all is indispensable for maintaining the ability of national health systems to provide universal health coverage.^1^ Nevertheless, worldwide, healthcare professionals tend to concentrate in urban areas, causing significant regional disparities in health services.^2^ Therefore, creating and sustaining an equitable level of medical personnel and resources in rural areas are critical components in building a national health system which can safeguard public health for all citizens.

Cambodia’s 16.3 million nationals have witnessed considerable improvements in public health over the last 20 years, with the country achieving most of the health-related Millennium Development Goals by 2015.^3^ Yet, in 2012, the nation had only 1.4 health workers per 1000 people,^4^ well below the critical shortage threshold defined by the World Health Organization (WHO). Some 40% of physicians and 74% of specialist physicians work in the capital city, Phnom Penh, whereas 80% of the population still resides in communes in rural areas and 90% of the nation’s poor are rural dwellers.^5^ This means that the regional inequity in health services remains a crucial problem, as illustrated by the fact that the infant mortality rate is approximately 3-fold higher in rural areas than in urban centes.^6^

Cambodia has both a regulated public health sector and an unregulated private health sector. Recent government policies have focused on improving health workforce numbers, infrastructure, capacity and worker distribution, particularly in the public sector.^7^ The nation’s public health systems operates through 8 national hospitals, 98 referral hospitals, and 1105 health centers (HC).^8^ Each referral hospital caters for 80 000-200 000 citizens, while each HC services 10 000.^8^ Medical staff who work in the HC are mostly nurses and midwives. HC staff supervise community health workers who are primarily non-medical staff.^8,9^ Thus, Ministry of Health has worked for increase and for much wider dispersion into rural areas of the number of functioning HC staffed according to Ministry of Health guidelines. Private hospitals and clinics are concentrated in urban areas, whereas HC cover residents in the remote countryside. There is tendency that HC provide health services for the poor while the private sector service the rich.^10^ Essentially, HC are the key to regional primary care, providing a wide range of essential health services. However, use of public providers remains relatively low for outpatient services.^3,6^ Sixteen percent of the ill or injured seek care first in the public sector, while 43% seek care from private providers.^3^ People visit private practitioners more for curative care, whereas preventive options, such as immunization, tuberculosis testing and HIV/AIDS prevention and control, are sought in the public sector.^3^ Consequently, Cambodia faces a prodigious challenge in establishing and maintaining a suitable public health sector workforce, including the provision of equipment, training and resources, human and financial.

Cambodia’s shortage of medical professionals is severe. Policies introduced have included medical education, regulation, and personal and professional support, with focus on the training of mid-level health workers such as nurses and midwives. But many challenges still remain. With respect to universities, private medical university are concentrated in urban areas, beyond the reach of rural poor students. To obtain appropriate knowledge, young medical staff need to be trained in well-equipped hospitals and specialist schools. Nevertheless, many healthcare workers in rural areas cannot afford to attend such centers due to lack of funds and opportunity. Moreover, working in urban private hospitals requires advanced English language, making them out of reach for many Cambodians. Besides, it is apparent that Cambodia is now suffering the twin threats of communicable and non-communicable diseases (NCDs). However, health workers in rural areas have limited opportunity to improve their knowledge and training on diagnosis, treatment and prevention especially toward NCD. Minatory of Health has established short and medium-term actions that the WHO Package of Essential Non-communicable Disease Interventions, single visit cervical screening, and palliative care are defined in the priority project to develop human resources for NCD,^11^ however the action plan has not completely been introduced to all HC. Actually, many HC mainly focus on only hypertension patients for prescription among NCDs patients. Furthermore, WHO package can stand further improvement to enhance the workforce toward NCDs in Cambodia, besides, education period and place are needed for palliative care, however the facility to learn palliative care tend to luck. Especially, practical training on patients might be necessary to enhance knowledge for NCD among medical staff, and policies for that purpose and facilities (eg, for radiography and laboratory) to perform diagnoses of NCD might be also required to enhance knowledge for NCD among medical staff.

After graduating, many young females nurse quickly give up working in rural areas. The salary for medical professionals working at a single medical facility is insufficient in rural locations. In addition, the work hours, stress and working environment are unforgiving compared to other professions. Comparatively speaking, small businesses provide acceptable salaries in rural locations. Social traditions also dictate that women should get married and raise their children rather than earn an income. Women thus need to be incentivized to continue working in rural health facilities. This will necessitate improvements in financial support and social factors.^12^ It will require the prospect of financial stability, respectability, job satisfaction, and recognizable career path for young staff. The remedy will also require a major investment in health facilities and training nationwide.

In Cambodia, it was announced by governments in 2016 that all health workers (in urban and rural areas) should be promoted to engage to the work which implied permanent jobs and better social welfare benefits.^13^ The Ministry of Health is investing in health and moving towards universal health coverage, reflecting the vision, goals and targets of the Sustainable Development Goals. The national health budget has almost doubled in real terms in the last five years and there has been significant progress in providing financial risk protection for the poor through measures such as Health Equity Funds and voucher schemes, with the former covering all HC. Nevertheless, challenges persist in access and quality of services, with high out-of-pocket expenditure and a growing and poorly regulated private sector. As a private hospital group which engage in the daily medical care for local patients and social service activity in rural area, one of strategy for full fill this gap might be to invest referral system between public sector and private sector which do not have strong connection rather than to increase investing more to communicable disease department. For all residents are covered essential health service, including emergency medicine and NCD at the nearest HC, the national budget should be invested for the improvement of HC service, which plays a vital role in caring for the health of rural residents who are hard to reach medical service.

## Ethical issues

Not applicable.

## Competing interests

All the authors report no proprietary or commercial interest in any product mentioned or concept discussed in this article.

## Authors’ contributions

All the authors have made a substantial contribution to this research. YK, MT contributed to writing the paper. All authors contributed to the study design, data collection, and coordination with local stakeholders.

## Funding

This research did not receive any specific grant from any funding agency in the public, commercial or not-for-profit sector.

## Authors’ affiliations

^1^Department of General Internal Medicine, Sunrise Japan Hospital Phnom Penh, Phnom Penh, Cambodia. ^2^Department of Public Health, Fukushima Medical University School of Medicine, Fukushima, Japan. ^3^Department of Medicine, University of Puthisastra, Phnom Penh, Cambodia. ^4^Department of Neurosurgery, Sunrise Japan Hospital Phnom Penh, Phnom Penh, Cambodia. ^5^Department of Pediatrics, Sunrise Japan Hospital Phnom Penh, Phnom Penh, Cambodia.
